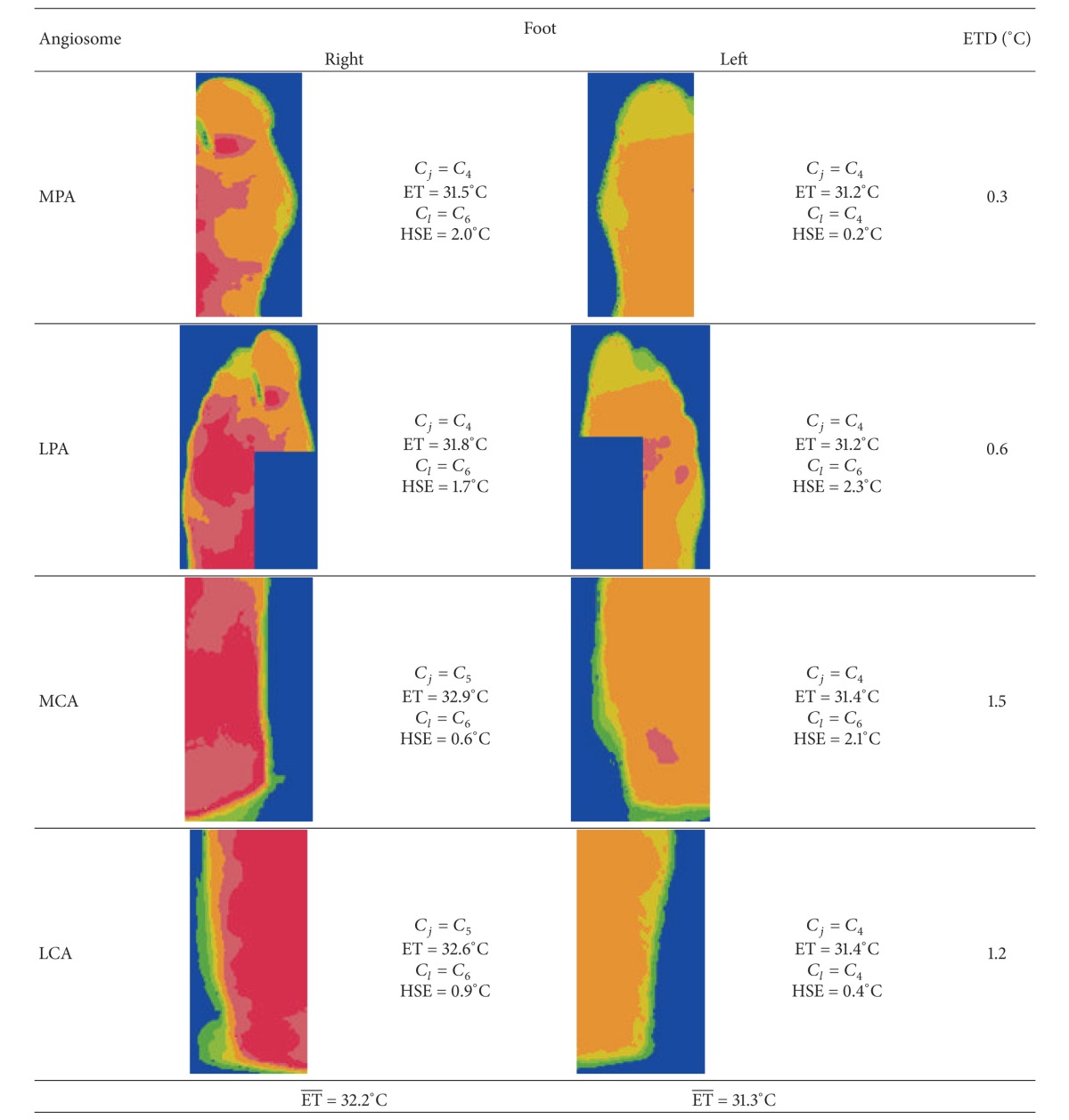# Quantitative Estimation of Temperature Variations in Plantar Angiosomes: A Study Case for Diabetic Foot

**DOI:** 10.1155/2014/585306

**Published:** 2014-02-13

**Authors:** H. Peregrina-Barreto, L. A. Morales-Hernandez, J. J. Rangel-Magdaleno, J. G. Avina-Cervantes, J. M. Ramirez-Cortes, R. Morales-Caporal

**Affiliations:** ^1^Instituto Nacional de Astrofísica, Óptica y Electrónica, Luis Enrique Erro No. 1, 72840 Tonantzintla, PUE, Mexico; ^2^Universidad Autónoma de Querétaro, Cerro de las Campanas S/N, 76010 Querétaro, QRO, Mexico; ^3^División de Ingenierías del Campus Irapuato-Salamanca, Universidad de Guanajuato, Carretera Salamanca-Valle de Santiago Km 3.5+1.8, Comunidad de Palo Blanco, 36885 Salamanca, GTO, Mexico; ^4^Instituto Tecnológico de Apizaco, Avenida Instituto Tecnológico, 90300 Apizaco, TLAX, Mexico

## Abstract

Thermography is a useful tool since it provides information that may help in the diagnostic of several diseases in a noninvasive and fast way. Particularly, thermography has been applied in the study of the diabetic foot. However, most of these studies report only qualitative information making it difficult to measure significant parameters such as temperature variations. These variations are important in the analysis of the diabetic foot since they could bring knowledge, for instance, regarding ulceration risks. The early detection of ulceration risks is considered an important research topic in the medicine field, as its objective is to avoid major complications that might lead to a limb amputation. The absence of symptoms in the early phase of the ulceration is conceived as the main disadvantage to provide an opportune diagnostic in subjects with neuropathy. Since the relation between temperature and ulceration risks is well established in the literature, a methodology that obtains quantitative temperature differences in the plantar area of the diabetic foot to detect ulceration risks is proposed in this work. Such methodology is based on the angiosome concept and image processing.

## 1. Introduction

Infrared (IR) technology allows the capture of natural heat radiation from the human body and its representation in a thermal image (thermogram) [1]. Since this heat radiation is produced by a thermal exchange process among skin tissue, inner tissue, local vasculature, and metabolic activity, the resulting temperature distribution could provide information regarding several diseases [1–3]. In diabetes mellitus cases, it is widely known that there is a high risk of complications in the foot; moreover, several studies have been focused in the analysis and characterization of its temperature. Some studies suggest a constant inspection of feet temperature in order to know how it behaves in diabetic patients [4, 5]. In addition, some reports have established that there are important differences of temperature in the plantar region between healthy and diabetic subjects with and without neuropathic damage [6, 7]. According to several investigations, the presence of hot regions in the plantar area may indicate tissue damage, inflammation, and arteriovenous shunting, which could compromise the nutritive capillary flow and increase the predisposition to cutaneous ulceration [8–10]. For instance, Brånemark et al. [11] found that diabetic patients present abnormal temperature patterns in feet and hands associated to common ulceration regions in a study made by using IR. Recently, Bharara et al. [12] proposed an analysis based on the thermal profile of a foot wound in order to provide a healing wound index for diabetic subjects. In fact, IR technology application has even been used to determine the required amputation level [13].

In [14], it is reported that nearly 50% of cases with diabetes mellitus have foot complications caused by a decrease of blood supply (vascular disorder) and loss of sensation (neuropathy). In the global scene, the incidence of foot ulceration is 2% in people with diabetes from which approximately the 15% will suffer the amputation of a limb [15]. This represents a major amputation every 30 seconds with over 2500 limbs lost per day [16]. Early detection of ulceration is complicated because diabetes causes a loss of connection between the muscle and the nerves (sensory denervation) and affects thermoreceptors and mechanoreceptors by depriving the patient from feeling any symptom, such as pain or swelling, when some injury has occurred [9, 12]. Diabetic patients require continuous medical care and demand the development of tools that bring reliable information so as to facilitate an early diagnostic [17]. Since the early detection of areas of risk on the diabetic foot is a topic of interest and temperature monitoring could reduce this risk of foot ulcerations and limb amputation [7], the development of methodologies that facilitate an opportune diagnosis turns into a relevant task. Therefore, it can be said that thermography represents an opportunity for early detection of ulcerations risk as well as a noninvasive and reliable method for diabetic foot care.

In this work, a methodology that determines and analyzes temperature differences is proposed to detect abnormal temperature increase in the diabetic foot. The plantar area is analyzed by taking into account the angiosome concept since the blood flow in these regions accurately reflects the temperature variation. In the angiosome, temperature estimation is calculated by identifying the present color regions, which at the same time are characterized and related to a temperature value. Thus, temperature differentiation between corresponding angiosomes can be achieved. Moreover, this process enables the localization of the area that covers each temperature; thereby, it also allows the detection of abnormal hot spots at the interior of the angiosome.

## 2. Materials and Methods

### 2.1. Patients Information

**Figure 1:**
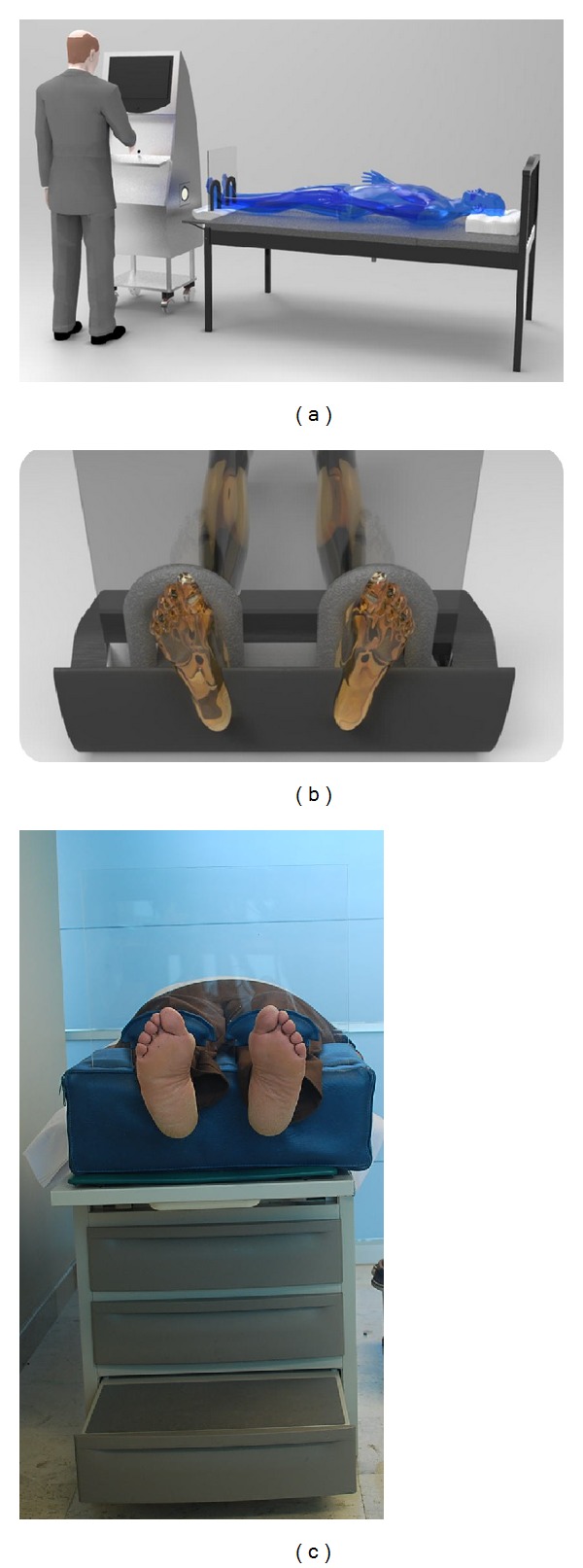
(a) The resting supine position for the image acquisition, (b) IR obstructive device, and (c) the preparation of real patient.

This study was carried out by considering a group of diagnosed patients with diabetes mellitus type 2 in the General Hospital of San Juan del Río, México, with the collaboration of a diabetes expert group in 2012. The focus group included male and female patients, in an age range from 35 to 80, with and without neuropathy. The patients attending a scheduled meeting were invited to participate in the study and they agreed. Exclusion occurred when the patient had an amputated toe, fractures or surgery of the lower limbs, peripheral arterial disease, presence of ulcers, or a history of ulcers. Thermograms were obtained in a conditioned room at controlled temperature of 25 ± 1°C where the patient is allowed to rest in supine position, as it is suggested in previous works [5, 13, 18]. The recommendations of the International Academy of Clinical Thermology [19] were followed as well. As underlined in previous works, this preparation allows the detection of skin temperature variations in the range of 0.05 to 0.1°C [2]. The preparation was done as follows: patient is asked to remove shoes and socks and to clean his/her feet with a damp towel. After that, patient is placed in supine position on the examination bed (Figure 1(a)), while the medical specialist collects some data (name, age, sex, height, weight, body temperature). At the end of the first 15 to 20 minutes of stabilization, an IR obstructive device is placed on patient feet in order to avoid outer heat radiation to appear in the thermogram (Figure 1(b)). The isolated feet temperature with its respective color palette and temperature scale is obtained by the thermogram.

The thermographic camera sensor is an IR detector, which absorbs the infrared energy emitted by an object and converts it into an electrical signal. IR is a technology that uses the blackbody radiation law, proposed by Max Plank, which establishes that every object with temperature above zero absolute emits electromagnetic radiation, also known as infrared radiation or thermal radiation [3, 20]. Later, Hardy [21, 22] proposed that human skin could be considered as a blackbody radiator, starting the IR use in medicine. In this way, when the surface of the skin changes, the emitted heat radiation is captured by the IR sensor and is translated into a thermogram. When a region of the skin increases/decreases its temperature, this region stands out from the background. Each pixel of a thermogram has a specific temperature value, and the image contrast is derived from the skin surface differences (gradients) in temperature. In this work, the thermograms have a color representation and were captured with an IR camera FLIR A300 with a thermal sensitivity of 0.05 at 30°C.

### 2.2. Color Characterization

**Figure 2:**
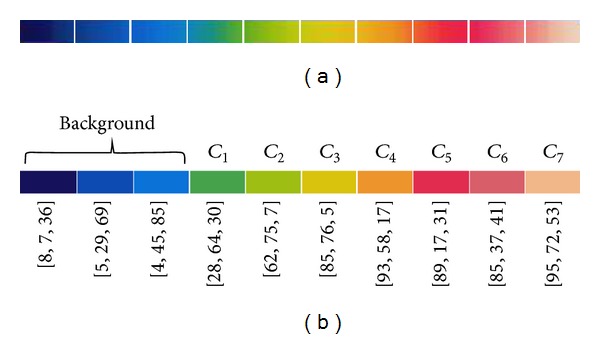
(a) Original color palette and (b) palette of representative colors with their respective RGB coordinates.

Thermograms are represented in the RGB color space, and a rainbow palette is used for representing a scale of 25–35°C. The value range was discretized into ten segments with the aim of representing 1°C per segment. However, the color palette is blurred and most of the segments have a soft transition between colors at the beginning (Figure 2(a)). This characteristic may complicate the definition of one representative color per segment; therefore, in order to avoid this problem, the median value was extracted from the spatial center of the segment. In this way, the extreme colors are not involved and the representative color is the most predominant among the colors contained in the segment.  Figure 2(b) shows the resulting palette with the representative color of each section, as well as their corresponding RGB coordinates.

Since the color identifies the temperature in a thermal image, each segment of the new palette has an associated value in centigrades (°C). The palette of representative colors now is simplified to eight representative color classes. The first three segments, from left to right in Figure 2(b), are taken as one single segment related to the coldest regions or background in the image with an interval of [25,28)°C. The background corresponds to a thermal insulation board used in the capture of images to avoid heat sources unrelated to the feet area (Figure 1(a)). The other segments have an individual representation and are associated with a temperature that varies in the range [28,35)°C, as shown in Table 1. This was determined by taking into account the results reported in [6], where subjects with diabetes risk have a temperature of 30.2 ± 1.3°C, and healthy subjects have a temperature of 26.8 ± 1.8°C, and after verifying that the mean plantar temperature of the patients was consistent with these ranges. In this way, it is possible to separate the feet area from the background. Since each segment of the palette represents an interval of the temperature, a representative classmark is assigned to them as shown in Table 1.

### 2.3. Temperature Estimated Difference

**Figure 3:**
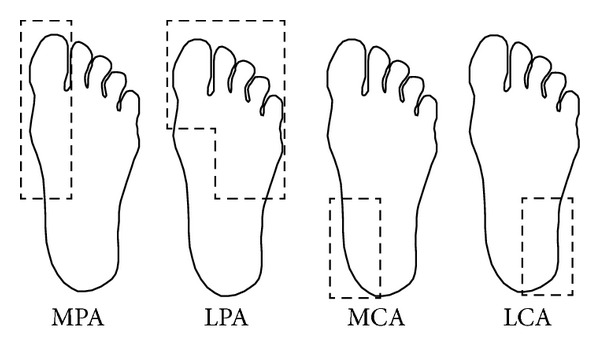
Angiosomes suggested by Taylor and Palmer [24] for temperature analysis.

In order to analyze the temperature distribution of the foot, it is necessary to establish a method for this analysis. In a recent study, Nagase et al. [23] suggested the use of the angiosome concept, proposed by Taylor and Palmer [24]. An angiosome in a foot is a region of tissue which has blood supply by a single artery. These regions are important since one of the main causes of diabetic foot ulceration is produced by a decrease of blood supply [5]. In 2006, Attinger et al. [25] proposed four main angiosomes as regions of interest in the plantar area for their temperature analysis (Figure 3): medial plantar artery (MPA), lateral plantar artery (LPA), medial calcaneal artery (MCA), and lateral calcaneal artery (LCA). Therefore, in this work the analysis of the plantar area is made based on the angiosomes concept and using the temperature estimation approach proposed by Peregrina-Barreto et al. [26]. The thermal image of each foot is divided into four subimages, corresponding to the four angiosomes mentioned before, and then all the pixels are classified according to their temperature. The image division is manually performed.

Let *I* ∈ *ℜ* ^*n*×*m*^ be an image corresponding to an angiosome, *p*_RGB_(*x*, *y*) ∈ *I* is a pixel in the color space RGB, *C* is the set of temperature classes, and *C*_RGB_ is the set of the RGB coordinates of each class. Consequently, the pixel classification is achieved through the following rule:

(1) $$\begin{array}{c} k\in \underset{C_{\text{RGB}}}{\text{argmin}}{||p_{\text{RGB}}-C_{\text{RGB}}||}_{2}, \end{array}$$

where *k* is the index related to the closest temperature class to *p*; that is, the *C*_*k*_ is the class assigned to *p*. An area value *A* = {*a*_0_,…, *a*_7_} has been assigned to each temperature class. When a pixel is classified with the class *C*_*k*_, its corresponding area (*a*_*k*_) is increased. In this way, when the complete image is processed, the area covered by certain class is known. In the case of the background, composed of more than one segment, all pixels are considered belonging to the same class (*C*_0_). The foot total area (*a*_total_) is composed of the area of all classes in *C*, except *C*_0_:

(2) $$\begin{array}{c} a_{\text{total}}=\overset{7}{\underset{i=1}{\sum }}a_{i}. \end{array}$$

In order to approximate the general temperature of an angiosome, it is considered the weighted mean of the largest class and its adjacent classes as the estimated temperature (ET),

(3) $$\begin{array}{c} \text{ET}=\frac{a_{j-1}C_{j-1}+a_{j}C_{j}+a_{j+1}C_{j+1}}{a_{j-1}+a_{j}+a_{j+1}}\;\text{s}.\text{t}\;j\in \underset{A}{\text{argmax}}\left(A\right), \end{array}$$

where *j* is the index of the element belonging to *A* with higher value. Since the estimated temperature of an angiosome is known, it is possible to have the estimated temperature difference (ETD) of the corresponding angiosomes between both feet to determine if the difference is normal [20]

(4) $$\begin{array}{c} \text{ETD}=\left|\text{E}{\text{T}}_{\text{left}\;\text{Angiosome}}-\text{E}{\text{T}}_{\text{right}\;\text{Angiosome}}\right|. \end{array}$$

### 2.4. Hot Spots Detection

Hot spots are considered small regions on the foot with a higher temperature compared to the ET value of the angiosome they belong. Previous works reported that hot spots occur mainly upon the metatarsal heads, great toe and heel, all of them areas of high foot pressure [8, 11]. Hot spots frequently lead in a high risk of ulceration thus their early detection is important. Considering that ETD value is based on the most representative areas, it does not bring information about the beginning of the temperature increasing (hot spots) that could represent a major risk when they cover a larger area. In order to determine if a hot spot represents an ulceration risk, a deeper analysis of the temperatures in the angiosomes is carried out. First, the higher index is selected (*l*) among the corresponding indexes of the elements of *A* with nonzero values,

(5) $$\begin{array}{c} l=\underset{X}{\text{argmin}}\left(X\right)\;\text{s}.\text{t}.\;X=\left\{i\;|\;a_{i}\in A\neq 0\right\}. \end{array}$$

As a result, the higher temperature present in the angiosome (*C*_*l*_) is known regardless the area that it covers. Thereupon, the presence of a hot spot is estimated by obtaining the difference of temperature between *C*_*l*_ and the ET (6). The hot spot estimator (HSE) provides useful information on whether there are small hot spots and how much they differ from the general temperature of the angiosome:

(6) $$\begin{array}{c} \text{HSE}=\left|C_{l}-\text{ET}\right|. \end{array}$$

## 3. Experimental Results

**Figure 4:**
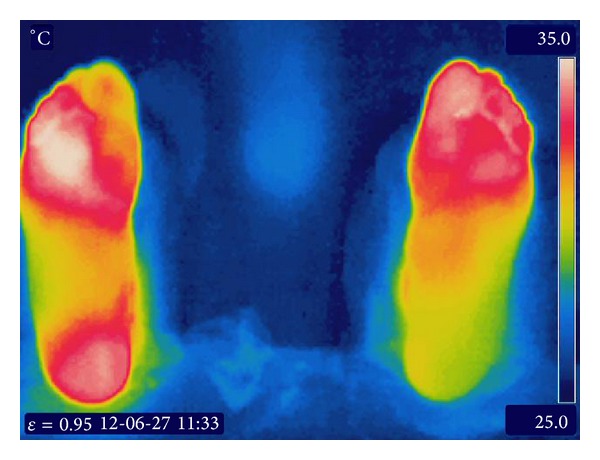
Original thermal feet image.

According to previous studies, the temperature variation between corresponding regions in both feet usually does not vary beyond 1°C; a difference greater than 2.2°C is considered as abnormal [15, 27]. The ranges established by [6] were considered in order to determine whether the plantar temperature is normal. Moreover, research results presented by Papanas et al. [7] about plantar temperature were taken into account. That study concluded that patients with diabetes type 2 with and without neuropathy have a plantar temperature of 32.2 ± 0.94°C and 30.7 ± 1.07°C, respectively. The goal in this work is to identify differences between corresponding areas of the right and left foot in order to obtain quantitative information about the plantar temperature distribution. Furthermore, it addressed the early detection of hot spots in an initial phase (small areas) with the aim of preventing their expansion and future complications. Both estimations could be useful for the specialist in early risk detection. The thermal images used in this work correspond to patients diagnosed with diabetes type 2 and neuropathy.  Figure 4 shows one of the obtained images, which is divided into two sections corresponding to the right and left foot. These images are processed in order to classify their pixels. All the pixels from each image are processed and classified in order to make the limits among the temperature regions clear. As it was described in Table 1, there are eight classes with a representative temperature and an associated color, and all the pixels are classified according to (1).

**Figure 5:**
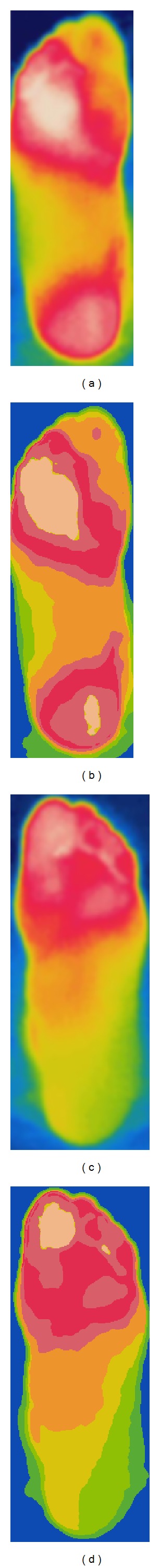
((a), (c)) Feet images extracted from Figure 4 and ((b), (d)) the result of pixel classification.

**Figure 6:**
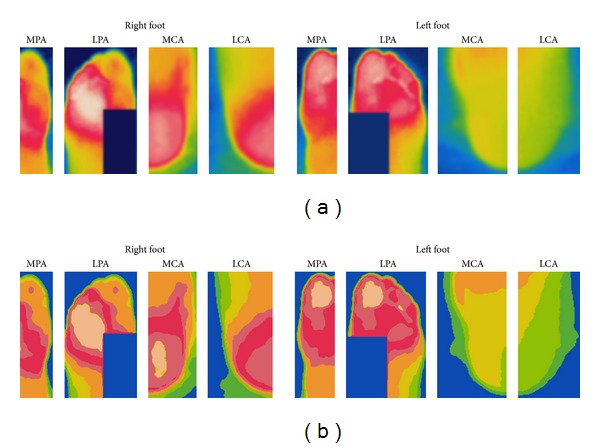
(a) Angiosome images of Figure 4 and (b) their color classification.

The importance of this process lies on the fact that in the original images it is difficult to determine where a color starts or ends due to the large number of colors (Figures 5(a) and 5(c)). The proposed classification process provides an estimated number of pixels (area) for every temperature class. Every time a pixel is classified as belonging to a class, the counter associated with that class is increased. Since only the foot area is relevant for the analysis, the remaining temperatures are considered background and they are not taken into account for the measurement. Thus, the classes *C*_1_ to *C*_7_ comprise the total foot area (*a*_total_). In this way, the foot is off the background, as it can be observed in Figures 5(b) and 5(d), where the background is uniform and the regions are well defined in their corresponding classes. In this case, the entire feet were processed by way of example and to provide a better perspective of the classification process. For practical purposes, in this methodology the pixel classification is as follows. Once the feet are separated in their corresponding images (Figures 5(a) and 5(c)), they are again divided into four subsections, one per angiosome (Figure 6). Observing the original MPA angiosome image of the left foot (Figure 6(a)), it can be supposed that the color corresponding to the *C*_5_ class has the greatest area. However, it is difficult to establish the limit among the colored regions in a clear way because of the similitude among some colors. A visual comparison between the MPA angiosome of both feet could be even more complicated; therefore, it is important to go beyond the visual perception and make a quantitative comparison of the temperature distribution. Once the pixel classification has been made, it is possible to clearly observe the area that covers certain temperature associated with a class.

**Figure 7:**
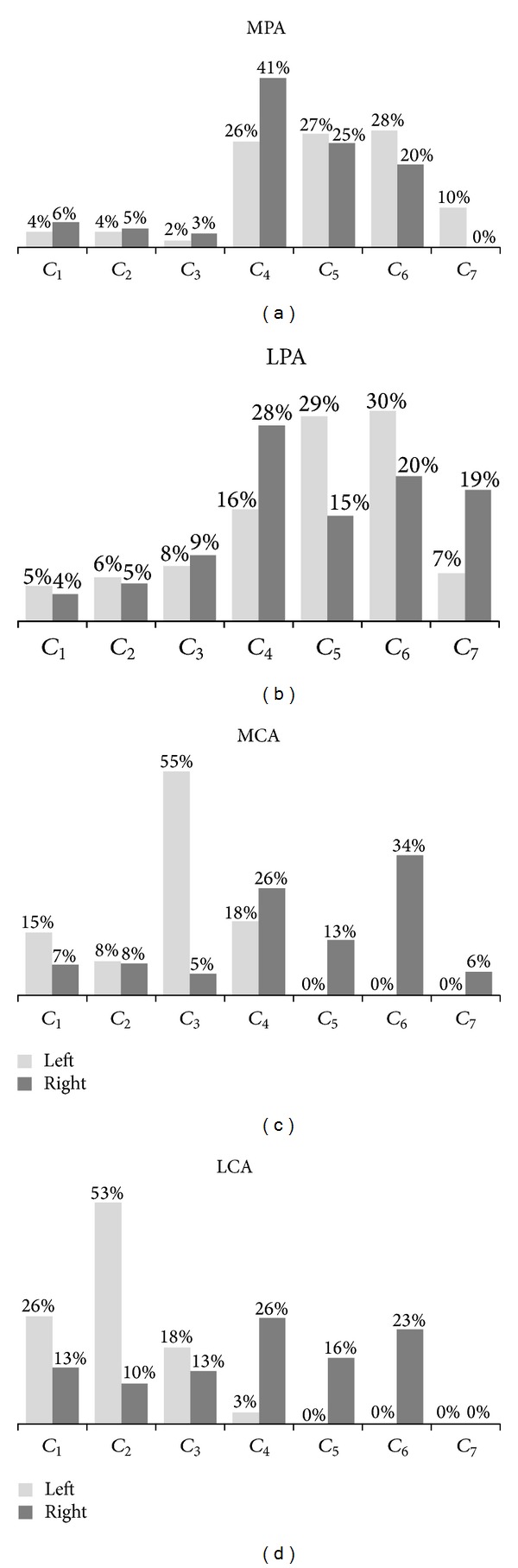
Graphical representation of the area (as percentage) that covers each temperature class in the angiosome (a) MPA, (b) LPA, (c) MCA and (d) LCA of the left and right feet of Figure 6(b). The representative temperature of an angiosome is given by the larger class and their two adjacent classes.

The areas of each angiosome in Figure 6(b) are shown in the plot of Figure 7. The areas are translated into percentages and based on this data, it determined which classes have the larger area in the same angiosome of both feet. In the MPA angiosome analysis, it is observed that the majority of the angiosome area is divided among classes *C*_4_, *C*_5_, and *C*_6_ for the left foot (Figure 7(a)), that is, a temperature interval of [31, 34)°C according to Table 1. For the right foot, the class *C*_4_ is the predominant one with an interval of [31, 32)°C. In the LPA plot (Figure 7(b)), it is observed that the foot temperatures are more distributed, being *C*_5_ and *C*_6_ the largest classes for the left foot, while *C*_4_ is the largest for the right foot. Then, the temperature intervals in the LPA angiosome are [32, 34) and [31, 32)°C for the left and right foot, respectively. For the calcaneal angiosomes (MCA and LCA), the temperature distribution has a more drastic variation respect to MPA and LPA. The MCA angiosome temperature in the left foot with a predominant class *C*_3_ is lower ([30,31)°C) than in the right foot with a predominant class *C*_6_ ([33,34)°C), as shown in Figure 7(c). LCA temperature is also very different, being *C*_2_ ([29, 30)°C) in the left foot and, *C*_4_ ([31, 32)°C) the right foot, the predominant classes (Figure 7(d)).

The importance of including the data of the adjacent classes is because they have values closer to the class with larger area and they allow a better estimation of the temperature by (3). For the MPA in the left foot, *C*_*j*_ = *C*_6_ and the adjacent classes are *C*_5_ and *C*_7_ with classmarks 33.5, 32.5 and 34.5 and areas 28, 27 and 10 (percentages), respectively. Thus, the estimated temperature in this region is ET_leftMPA_ = 33.2°C. Following the same procedure, the MPA for the right foot with *C*_*j*_ = *C*_4_ has an ET_rightMPA_ = 31.8°C. With these data, it is possible to estimate by (4) that between the MPAs regions there is an ET_DMPA_ = 1.4°C.  Table 2 summarizes the described estimation for all the angiosomes of both feet by (3). In Figure 6(a), it is noted that the biggest visual difference is in the MCAs and LCAs angiosomes, and after computing their data it is known that these regions have a difference of temperature that exceeds the normal difference of 2°C. In addition, if the mean temperature of the whole feet ($\bar{\text{ET}}$) is estimated based on the temperatures of each angiosome, then both feet have a temperature of 31.6°C and 32.1°C, respectively. The difference between this data suggests a small difference in the temperature condition of the feet. However, after a deeper analysis, it can be observed that there are important differences in some regions that may not be distinguished in a qualitative analysis.

**Figure 8:**
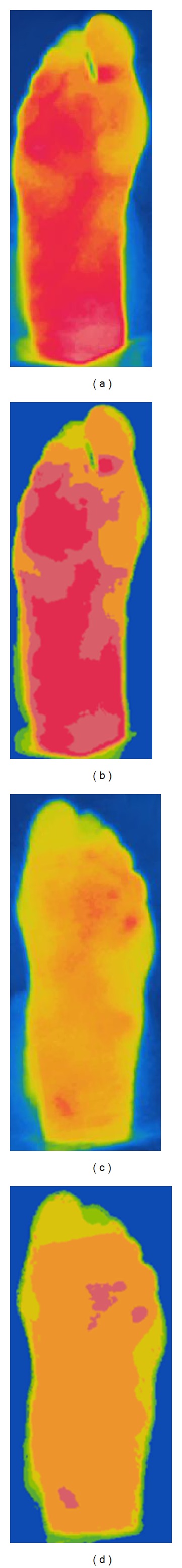
Example 2 of thermal feet images ((a), (c)) and their results after pixel classification.

Another example is presented in Figure 8, where it is noticed how the pixel classification could be useful in the improvement of the temperature regions limitation.  Figure 8(a) presents a wide red region in which it is difficult to define the limits of a color due to smooth transitions. Some regions even seem to be homogeneous at first sight. However, after the classification process (Figure 8), the regions identification is clearer and their analysis is easier; it is noted the presence of small hot spots.  Table 3 summarizes the information obtained from the temperature classes and areas. As it is observed, the most representative temperature classes are similar in some angiosomes in which ETD has lower values. According to the ETD values, there are no abnormal differences between feet; nevertheless, it is important to analyze the temperature differences inside an angiosome with the aim of detecting an additional type of abnormalities: hot spots. Although ETD brings important information, it does not detect abnormalities when the temperatures in the feet are similar because it is based on the values of larger classes. As mentioned before, a hot spot is an area whose temperature is significantly higher respect to its adjacent areas and the HSE value tries to detect it in an initial phase. Hot spots detection provides information that help to detect suspicious areas where an ulceration process could be starting.

In order to detect hot spots, the HSE value is calculated by (6). First, it is necessary to determine the class associated with higher temperature present in an angiosome (*C*_*l*_). For instance in the MPA angiosome of Figure 8, *C*_*l*_ corresponds to *C*_6_ for the right foot then, HSE value is 2.0°C right foot; once again, the differences in more than 2°C degrees are suspicious. In this angiosome, a small hot spot near the thumb bottom as well as a wider area belonging to *C*_4_ can observed. The HSE value suggests that there are abnormal areas but it is the expert who should decide which ones must be under observation. This is because an angiosome only shows a segment of the plantar area and one region detected as a hot spot may be part of a larger region and not a hot area in an initial phase. This is the case for the hot spots detected in the right MPA. For the left MPA with *C*_*l*_ = *C*_4_, the HSE value is 0.2°C. In the analysis of the LPA angiosome (which include the MPA region mentioned above) the HSE does not show abnormal differences in the right foot. For the left LPA the detector indicates a difference of 2.3°C which is associated with three hot spots near each other. In this case, the hot spots neither belong to a larger region nor appear in the respective MPA. Another hot spot was found in the left MCA where *C*_*l*_ = *C*_6_ and the HSE = 2.1°C. Although the angiosome differences (ETD) do not indicate abnormal temperatures, the HSE estimator indicates the presence of possible hot spots in the right MPA, and in the left LPA and MCA. The estimator could be suggesting that these angiosomes contain suspicious areas which should be under a continuous revision.

It is observed that the mean temperatures of the feet in Figure 5 (31.6°C and 32.1°C) and Figure 8 (32.2°C and 31.3°C) exceed the normal plantar temperature (26.8 ± 1.8°C) and both are in the characteristic range classified as neuropathy (32.2 ± 0.94°C). It can be noticed by observing the temperatures of the angiosomes that most of them are also in this last range. Both thermographs correspond to patients diagnosed with neuropathy as mentioned before.

## 4. Discussion

While there is an important amount of scientific reports about temperature analysis in the diabetic foot, most of them rely on qualitative analysis. However, it is not always simple to estimate the abnormal temperature differences by visual inspection of the thermogram. The aim of the proposed methodology is to bring precise information about such differences by facilitating the detection of possible risks regions and their evolution to the medical specialist. Although not all the regions with abnormal temperature will become an ulcer, their monitoring is very important as they represent high-risk regions. It is significant to remark that this methodology is not a diagnostic tool but a tool that brings complementary information to be evaluated by the medical specialist in order to facilitate early detection of ulceration risks.

## 5. Conclusions

Thermal imaging and image analysis are useful tools in the medicine field applied to the study of diseases such as diabetes. Temperature distribution in the plantar area contains relevant information about the condition of diabetic foot and ulceration risks. In this work, a methodology was presented aiming at providing quantitative information about abnormal temperature differences in symmetric regions between feet and inside of the same foot. The methodology took into account the temperature differences, their distribution, and area. As a first analysis, differences between symmetric areas in both feet were studied since it is known that symmetric regions in the body have similar temperatures. For this, the plantar area was divided into four main regions (angiosomes) and the temperatures inside those regions were grouped in classes according to a color similitude criterion. An index (ET) based on the relation between the class with larger area and its adjacent classes was proposed in order to estimate a representative temperature for each angiosome. Thus, it was possible to obtain an estimated difference (ETD) between symmetric regions which obtained an accurate measure to determine whether there is an abnormal difference or not. A second analysis was performed to study the temperatures inside the angiosomes with the aim of detecting the presence of small abnormal areas (hot spots). For that reason, it proposed a hot spot estimator (HSE) that relates the representative temperature of the angiosome with the higher temperature on it. This estimator was capable to detect the presence of abnormal regions in initial phase that, for their small area, were not detected by the estimator ETD. In this way, it was possible to analyze the whole plantar area by bringing quantitative information to determine the presence of regions in possible risk of ulceration. The results of the temperature measurement agreed with previous reports about the diabetic foot temperature characterization. Thus, this study provided an approach to bring reliable information to help the specialist in the early detection of the ulceration risks of the diabetic foot associated with high temperatures.

## Figures and Tables

**Table 1:** Temperature classes.

Temperature classes	Interval (°C)	Classmark (°C)
*C*_0_	[25, 28)	26.5
*C*_1_	[28, 29)	28.5
*C*_2_	[29, 30)	29.5
*C*_3_	[30, 31)	30.5
*C*_4_	[31, 32)	31.0
*C*_5_	[32, 33)	32.5
*C*_6_	[33, 34)	33.5
*C*_7_	[34, 35)	34.5

**Table 2:** Temperature analysis of Figure 4.

Angiosome	Foot	*C*_*j*_	ET (°C)	ETD (°C)
MPA	Left	*C*_6_	33.2	1.4
	Right	*C*_4_	31.8	
LPA	Left	*C*_6_	33.1	1.5
	Right	*C*_4_	31.6	
MCA	Left	*C*_3_	30.6	2.8
	Right	*C*_6_	33.4	
LCA	Left	*C*_2_	29.4	2.2
	Right	*C*_4_	31.6	

**Table 3:** Temperature analysis of Figure 8.